# Accelerated forgetting in presymptomatic Alzheimer’s: mediation by prefrontal cortical degeneration

**DOI:** 10.1093/braincomms/fcaf478

**Published:** 2025-12-09

**Authors:** Christopher S Parker, Chloe Young, Nicholas Magill, Kirsty Lu, Sebastian J Crutch, Nick C Fox, Philip S J Weston

**Affiliations:** Dementia Research Centre, UCL Queen Square Institute of Neurology, London, WC1N 3AR, UK; UCL Hawkes Institute, Department of Computer Science, London, WC1V 6LJ, UK; Dementia Research Centre, UCL Queen Square Institute of Neurology, London, WC1N 3AR, UK; Dementia Research Centre, UCL Queen Square Institute of Neurology, London, WC1N 3AR, UK; London School of Hygiene and Tropical Medicine, Medical Statistics, London, WC1E 7HT, UK; Dementia Research Centre, UCL Queen Square Institute of Neurology, London, WC1N 3AR, UK; Dementia Research Centre, UCL Queen Square Institute of Neurology, London, WC1N 3AR, UK; Dementia Research Centre, UCL Queen Square Institute of Neurology, London, WC1N 3AR, UK; UK Dementia Research Institute at UCL, London, NW1 3BT, UK; Dementia Research Centre, UCL Queen Square Institute of Neurology, London, WC1N 3AR, UK; UK Dementia Research Institute at UCL, London, NW1 3BT, UK

**Keywords:** accelerated long-term forgetting, Alzheimer’s disease, prefrontal cortex, cortical thickness, memory consolidation

## Abstract

In Alzheimer’s disease (AD), accelerated long-term forgetting (ALF), where information is retained normally over 10–30 min but lost at an accelerated rate over subsequent days to weeks, develops several years before symptom onset. However, the neuroanatomical changes underpinning ALF remain undetermined. Eighteen presymptomatic autosomal dominant AD mutation carriers and 12 non-carriers underwent ALF assessment with a list, a story, and visual figure, testing 30-min and 7-day recall of each, separately. T_1_ and diffusion-weighted MRI were acquired. Cortical thickness was estimated for 13 pre-defined grey matter regions, with streamline tractography assessing associated structural connectivity. In mutation carriers, lower verbal ALF performance (list and story) was strongly associated with thinner prefrontal cortex (PFC) across four contiguous regions bilaterally. This association was absent in non-carriers. No associations were found between ALF and the thickness/volume of medial temporal lobe (MTL) structures. The association between ALF and PFC connectivity was weaker than for cortical thickness. Our results suggest that early subtle pathological change in PFC underpins ALF development, highlighting the central role of PFC dysfunction in very early AD-related cognitive decline. ALF may represent a qualitatively different (non-MTL driven) form of forgetting compared with the short interval forgetting that develops at later disease stages.

## Teaser

Parker *et al.* find that accelerated long-term forgetting in familial Alzheimer’s disease is mediated by neurodegeneration in the prefrontal cortex and not medial temporal structures or white matter connections. This suggests the neuroanatomical process underlying early long-term memory deficits in Alzheimer’s disease is qualitatively different to later short interval memory impairment.

## Graphical Abstract

**Graphical Abstract:**
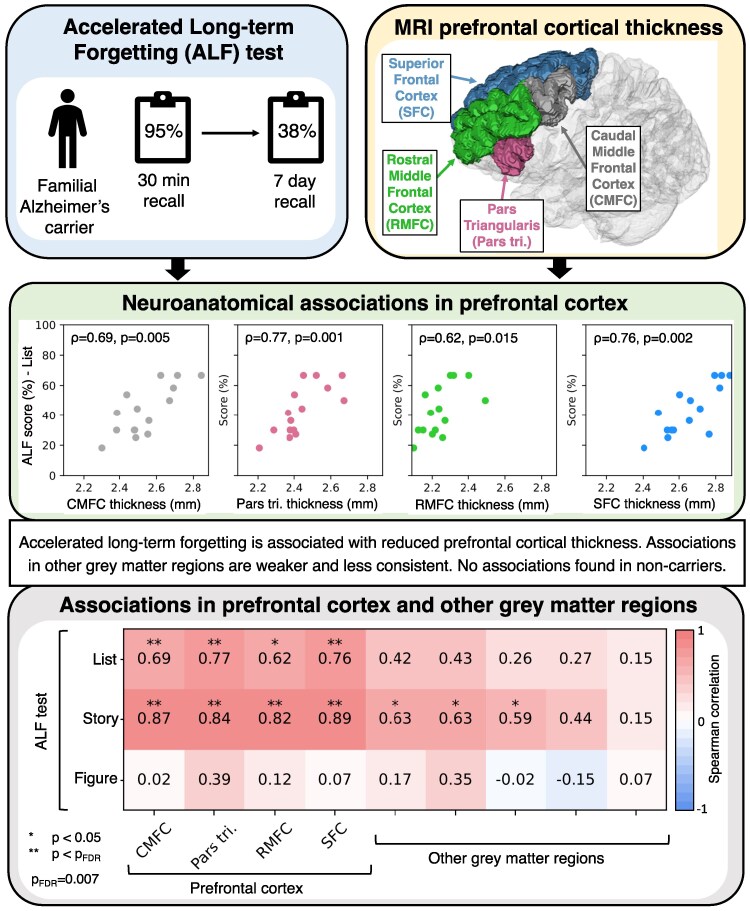

## Introduction

In Alzheimer’s disease (AD), subtle cognitive decline begins several years before the onset of overt clinical symptoms,^1^ but understanding of the precise neuroanatomical basis of the earliest changes remains limited. While it is widely accepted that episodic memory is the first cognitive domain affected in AD, conventional memory tests, assessing recall over intervals of 5–30 min, lack sensitivity to early, presymptomatic change.^2^

Accelerated long-term forgetting (ALF) is a pattern of memory dysfunction where encoding and initial storage of new information occurs normally but is then lost at an accelerated rate over the following days and weeks, and is thought to represent early breakdown in long-term consolidation.^3^ We previously found that ALF was a feature of presymptomatic AD by assessing a cohort of autosomal dominant AD (ADAD) mutation carriers, with mutations in either presenilin 1 or amyloid precursor protein genes.^4^ ADAD shares many features, both pathophysiologically and clinically, with the more common sporadic form, and allows prospective study of asymptomatic individuals with known AD pathology.^5^ Presymptomatic mutation carriers, who were on average an estimated seven years from expected symptom onset, had an increased rate of forgetting over a seven-day period despite demonstrating normal learning, normal 30-min recall, and normal performance on a comprehensive battery of other cognitive tests.^4^ These findings have been replicated in multiple presymptomatic sporadic AD cohorts,^6-8^ with ALF potentially representing the earliest detectable cognitive change in AD.^9^

While there is increasing recognition of the development of ALF in presymptomatic AD, its neuroanatomical basis has remained uncertain. Focal correlates of impaired short interval recall, as occurs in mild cognitive impairment and AD dementia, are well characterized, being shown repeatedly to be associated with focal changes in medial temporal lobe (MTL) volume.^10,11^ However, to date, neuroimaging studies in both AD and temporal lobe epilepsy have found no consistent association between MTL structural measures and ALF,^4,8,12^ with the relative influence of medial temporal versus neocortical structures a topic of ongoing debate.^13^ A further possibility, previously unexplored, is that the development of ALF is also influenced by changes in connectivity, with AD known to involve loss of white matter connectivity across a network of grey matter nodes.^13^

We undertook a multimodal MRI study with the aim of characterizing the anatomical correlates of ALF in presymptomatic ADAD, both in terms of grey matter thickness/volume and structural connectivity.

## Material and methods

### Participants and cognitive data collection

A total of 30 asymptomatic individuals from families affected by ADAD were included: 18 mutation carriers (who therefore have presymptomatic AD pathology) and 12 non-carrier controls. All participants underwent assessment with the Clinical Dementia Rating Scale and a comprehensive battery of additional cognitive tests.^4,14^ Participants were assessed on initial learning, 30-min recall, and 7-day recall of (i) a word list, (ii) a short story, and (iii) a complex visual figure, as previously described (also see Supplementary material).^4^ The ALF score was calculated by dividing the 7-day recall score by the 30-min recall score and converting to a percentage, with higher scores representing better long-term retention.

### MRI acquisition

MRI scans were acquired on a 3-Tesla Siemens TIM Trio scanner using a 32-channel phased array head-coil. A sagittal 3D MP-RAGE T_1_-weighted volumetric MRI (echo time/repetition time/inversion time = 2.9/2200/900 ms, dimensions 256 × 256 × 208 mm, voxel size 1.1 mm isotropic) was acquired. A 64-direction diffusion-weighted image (DWI) sequence was acquired with a single shot echo planar imaging (EPI) sequence (field of view 240 × 240 mm; matrix 96 × 96; voxel size 2.5 mm isotropic; 55 contiguous axial slices; repetition time 6800 ms; echo time 91 ms; *b* value 1000 s/mm^2^). Nine volumes were acquired without diffusion weighting (*b* = 0 s/mm^2^). The median time between ALF testing and MRI scanning was 13 weeks (inter-quartile range was 12 weeks).

### T1-weighted image processing and analysis

**Figure 1:**
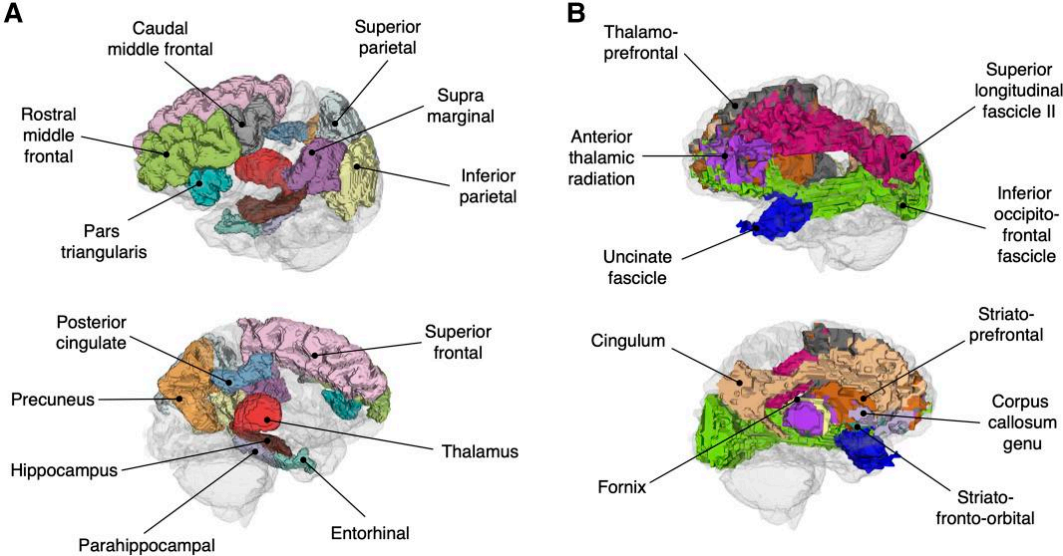
**Grey matter and white matter ROIs.** (**A**) Cortical and sub-cortical grey matter ROIs, selected based on either (i) having a known vulnerability to early AD-related neurodegeneration,^17,18^ or (ii) having a known role in normal memory function.^15,16^ (**B**) White matter ROIs used in the second part of the study, with choice informed by the results of the grey matter analysis.

**Figure 2:**
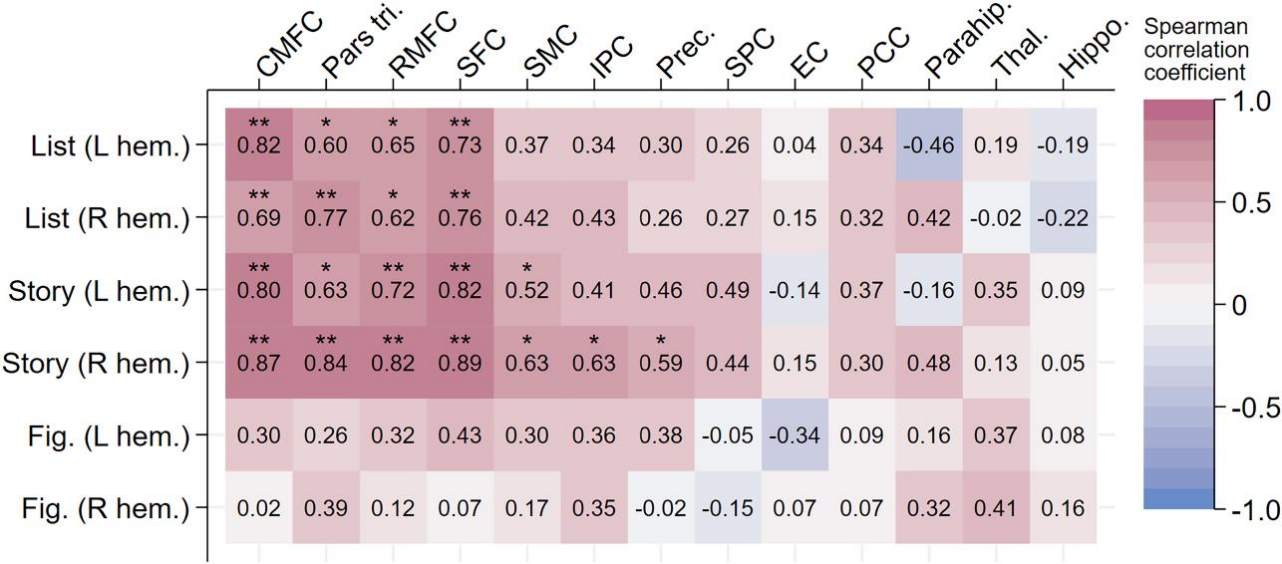
**Association between focal grey matter thickness/volume and ALF scores in mutation carriers.** Results for Spearman correlation coefficients between ALF list, story and figure scores (split by left and right hemisphere) and each region of interest, with statistically significant associations indicated. Correlations were analysed in *n* = 18 mutation carriers. Consistent focal associations are seen between verbal ALF and cortical thickness across the prefrontal cortical ROIs (details of exact coefficients and *P*-values can be found in Supplementary Table 1). CMFC, caudal middle frontal cortex; EC, entorhinal cortex; Fig., Figure; hem., hemisphere; Hippo., hippocampus; IPC, inferior parietal cortex; L, left; Parahip., parahippocampus; Pars tri., pars triangularis; PCC, posterior cingulate cortex; Prec., precuneus; R, right; RMFC, rostral middle frontal cortex; SFC, superior frontal cortex; SMC, supramarginal cortex; SPC, superior parietal cortex; Thal., thalamus. Correlation *P*-values were calculated using a two-tailed *t*-test. Black stars indicate statistical significance (* *P* < 0.05, ** *P* < p_FDR_; p_FDR_ =0.00769).

Cortical and sub-cortical grey matter regions were parcellated from the T_1_-weighted images using FreeSurfer v5.30 (surfer.nmr.mgh.harvard.edu), with estimation of regional cortical thickness and sub-cortical volumes (Fischl and Dale, 2000). Thirteen grey matter regions of interest (ROIs), comprising both cortical and sub-cortical structures, were selected from the Desikan-Killiany atlas based on either (i) having a recognized involvement in normal episodic memory function,^15,16^ or (ii) being part of the ‘ADAD cortical signature’, a group of cortical regions known to be particularly vulnerable to early ADAD-related neurodegeneration^17,18^ (Figs. 1A and 2).

### DWI image processing and analysis

Following the analysis of associations between ALF and grey matter structures, we also investigated white matter structural connectivity between grey matter regions.

DWIs were corrected for susceptibility, motion and eddy current distortions. TractSeg^19^ was applied to parcellate the white matter into 10 bilateral tract ROIs (Fig. 1B), with ROI selection informed by the results of the previous grey matter analysis. Voxels with low diffusion tensor fractional anisotropy (<0.25) were removed to improve the specificity of TractSeg masks to the centre of each tract.

A white matter mask was delineated from the T_1_-weighted Freesurfer parcellation and aligned with the DWI using linear and non-linear registration. Streamlines seeded randomly throughout the white matter mask followed the local fibre orientation distribution (FOD), derived using constrained spherical deconvolution in MRTrix.^20^ Streamlines terminated after exiting the mask or encountering FOD amplitude < 0.1, and those < 12.5 mm were excluded. Streamlines were continually seeded until 200 000 viable streamlines were generated for each individual. The streamline density (SD) in each voxel was calculated and averaged over voxels within each ROI, to serve as a proxy measure of tract axon density.

### Statistical analysis

Spearman correlation coefficients were calculated to assess the association between ALF scores and imaging measures across first grey matter and subsequently white matter ROIs. This rank-based approach can be used with bounded variables and is robust to non-normality and outliers. Correction for multiple comparisons was performed using a False Discovery Rate (FDR) of 0.05.

Post-hoc comparison of the median cortical thickness of mutation carriers and non-carriers was performed using Mann-Whitney U-tests in order to aid interpretation of results.

## Results

Demographics and clinical data for participants are outlined in Table 1.

**Table 1:** Participant demographics and clinical data

	Mutation carriers (*n* = 18)	Non-carriers (*n* = 12)
Female (***n***; %)	8 (44.4%)	8 (66.7%)
Age (years)	37.43 (5.30)	38.77 (7.53)
EYO (years)	7.29 (4.72)	NA
Years of education	13.61 (2.59)	14.67 (2.23)
Global CDR	0 (0 to 0)	0 (0 to 0)
MMSE (out of 30)	29.00 (29.00 to 30.00)	29.50 (29.00 to 30.00)
List ALF (%)	43.06 (30.00 to 61.54)	71.79 (58.55 to 81.82)
Story ALF (%)	66.67 (58.82 to 83.02)	90.70 (78.80 to 96.71)
Figure ALF (%)	72.41 (62.75 to 83.88)	87.55 (75.68 to 96.25)

CDR, Clinical Dementia Rating Scale; EYO, estimated years to symptom onset (based on parental history); MMSE, Mini Mental State Examination. For age, EYO and years of education, means and standard deviations are shown. For global CDR, MMSE and ALF scores, medians and inter-quartile ranges are shown. ALF scores are calculated by dividing the 7-day recall score by the 30-min recall score and converting to a percentage, with higher scores representing better long-term retention.

Group differences in ALF between presymptomatic mutation carriers and non-carriers have been described previously, with mutation carriers demonstrating significantly worse ALF for list, story and figure recall tasks (Table 1, Supplementary Table 4), despite normal learning and 30-min recall, and normal performance on other cognitive tests.^4^

### Verbal ALF is associated with prefrontal cortical thickness in ADAD mutation carriers

**Figure 3:**
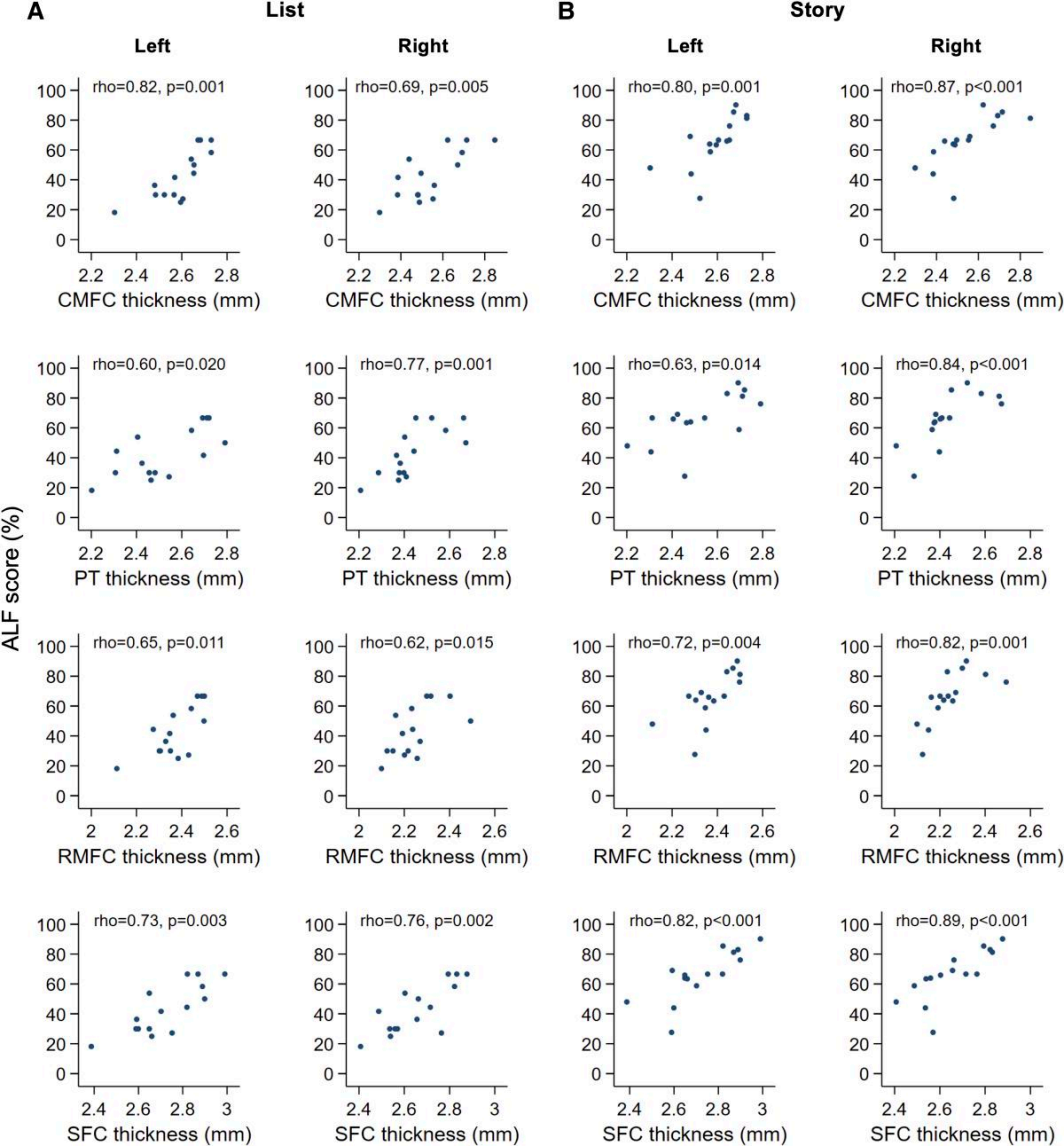
**Scatter plots of verbal ALF scores and cortical thickness in the PFC in mutation carriers.** Scatter plots are shown for (**A**) list ALF, and (**B**) story ALF across the four PFC ROIs for both left and right hemispheres separately. Each scatter plot shows the ALF score (*y*-axis) and cortical thickness/volume (*x*-axis) across *n* = 18 mutation carriers. Spearman’s rho and the associated *P*-value (uncorrected) are shown for each plot. CMFC, caudal medical frontal cortex; PT, pars triangularis; RMFC, rostral middle frontal cortex; SFC, superior frontal cortex. All *y* axes represent ALF score (7-day recall/30-min recall, expressed as a percentage).

In mutation carriers, there were strong and statistically significant associations between more severe verbal ALF (both list and story) and lower baseline cortical thickness in the prefrontal cortex (PFC) across four contiguous regions bilaterally (Figs. 2, 3, Supplementary Table 1). In mutation non-carriers, these associations were weak, with no evidence of statistical significance. In mutation carriers, there was no evidence of association between ALF and the thickness/volume of MTL structures (hippocampus, parahippocampus and entorhinal cortex) (Fig. 2). Any associations between verbal ALF and other grey matter ROIs were weaker and less consistent. No evidence of association was found between ALF figure score (i.e. visual ALF) and grey matter thickness/volume. The association between verbal recall and regional grey matter thickness/volume was present only for ALF (i.e. forgetting rates over 7 days), with no association found between 30-min recall and grey matter structure in any ROI (data not shown). Most of the associations between verbal ALF and PFC thickness survived multiple comparison correction (Fig. 1).

There was no evidence of group differences in cortical thickness in prefrontal ROIs (*P* > 0.15 for all comparisons, Supplementary Table 3), or any other ROIs, between mutation carriers and non-carriers (see Supplementary material), despite only mutation carriers demonstrating an association between verbal ALF and cortical thickness.

### The association between verbal ALF and prefrontal white matter connectivity is weaker than for cortical thickness

Following the results of the grey matter analyses, we undertook a hypothesis-driven investigation of white matter structural connectivity, aiming to assess whether in mutation carriers ALF is associated not only with prefrontal grey matter cortical structure, but also with the connectivity of PFC with other cortical and sub-cortical structures, including the MTL (Fig. 1B). Correlation coefficients for both list and story were consistently positive across all 19 ROIs (including left and right hemispheres separately), although reaching statistical significance in only three ROIs in total (list ALF with left cingulum, and left and right striato-prefrontal tracts), none of which survived multiple comparison correction (Supplementary Table 2). There were no statistically significant associations between SD and figure ALF. As with the grey matter analysis, there were no significant associations between 30-min recall (list, story or figure) and SD in any ROI, with the direction of the coefficients much less consistent (data not shown). There was no association between PFC structural connectivity and ALF in the non-carriers.

## Discussion

Our results demonstrate a strong and consistent association between verbal ALF and the structural integrity of the PFC in presymptomatic ADAD mutation carriers. This finding appears to be specific to mutation carriers (i.e. those with presymptomatic AD pathology) only, with no association found in non-carriers, despite there being no detectable difference between the two groups on conventional cognitive tests or cortical thickness. The finding is also specific to ALF, with no pattern of associations found for shorter interval recall. No association was found between verbal ALF and hippocampal volume, consistent with previous studies,^4,8,12^ with early development of ALF appearing to be mediated specifically by PFC.

The PFC performs a number of functions in cognition and behaviour, including decision making, memory retrieval and memory consolidation.^21,22^ Most of the AD-related neuron-specific transcriptional changes that occur in the PFC have been found to happen in the early, presymptomatic disease period, when amyloid is present but prior to significant tau deposition.^23^ Additionally, it has been shown that, when compared with other brain regions, PFC synaptic connections are particularly vulnerable to exposure to amyloid-β, undergoing early loss of dendritic spines and associated upregulation of synaptic gene expression, which may explain its influence on the early disruption of memory function.^24^

While we found a strong association between verbal ALF and PFC grey matter integrity, the evidence of an association between ALF and PFC structural connectivity was weaker. Although, for both verbal ALF tests, correlation coefficients were numerically positive in every ROI bilaterally (38/38), this reached statistical significance in only three, without a clear predominance for any particular connection, and did not survive multiple comparison correction. The consistency of the positive direction of the coefficients for list and story is suggestive of there being a true association, but the lack of statistical significance at the level of individual tracts in most cases would indicate that any relationship between ALF and PFC structural connectivity is weaker than that for grey matter cortical thickness. Therefore, while PFC white matter connectivity may potentially influence to some extent the development and severity of ALF, it would appear that early focal alteration in the structure and function of the cortex is the more dominant factor.

As noted above, while the association between verbal ALF and PFC cortical thickness, and to a lesser extent between verbal ALF and PFC connectivity, was only found in ADAD mutation carriers, there was no demonstrable difference in PFC thickness or connectivity between the mutation carrier and non-carrier groups. One potential explanation for this is that, while the thickness and SD between groups is the same, potential early synaptic dysfunction in mutation carriers (not detectable on MRI) may mean that they become more reliant on the *number* of neurons present to compensate for the dysfunctioning of individual neurons, causing an association between cortical thickness and ALF that is not present for non-carriers where synaptic function remains in-tact. Further study assessing associations between ALF and imaging techniques such as synaptic positron emission tomography, or with synaptic fluid biomarkers, will be valuable in further investigating this.

Our results may help to clarify the mechanistic underpinnings of ALF. Two opposing views have previously been proposed based on different theoretical models of memory consolidation, one that views ALF as *qualitatively* different from forgetting over shorter intervals^13^ and one that views it as a *quantitatively* less severe form of the same type of memory deficit.^25^ The argument for a qualitative distinction between early and late forgetting is based on the *standard model*,^26^ which proposes that consolidation of episodic memory involves two discrete stages: initial *fast* consolidation, mediated by the MTL (particularly hippocampus), followed days or weeks later by *slow* consolidation, where hippocampal-neocortical (primarily PFC) connections are replaced by cortical-cortical connections.^27^ Conversely, those who propose a quantitative difference, in line with the multiple trace theory,^28^ argue that the MTL remains centrally involved even over extended intervals. Our results, showing the influence of PFC thickness but not MTL thickness/volume, combined with previous studies also showing no association with MTL volume, provides findings supportive of the standard model, with the neuroanatomy underpinning ALF being qualitatively different to that of shorter interval forgetting.

While we found associations in mutation carriers between measures of focal brain structure and verbal ALF (both list and story), this was not the case for visual ALF. Therefore, the neuroanatomical correlate of visual ALF, and any similarities or differences to that of verbal ALF remain uncertain. In presymptomatic AD, ALF in the visual domain has been found to be less severe than in the verbal domain, which may explain the lack of identifiable association, although we would hypothesize that the anatomical basis would be similar.^4^ Another potential explanation relates to previous findings that the PFC tends to engage contextual information relevant to memories, which may be more available for the semantically meaningful verbal stimuli compared with the more abstract figure recall task.^21^ Future visual ALF assessment using more semantically salient stimuli may help to address this question.^29^

Our study has limitations. The sample size is relatively small, primarily because of the relative rarity of ADAD, although was still sufficient to identify strong and consistent associations within the mutation carrier group. Replication in both familial and sporadic presymptomatic AD cohorts will be important. We did not collect data on participant’s sleep. Sleep is known to play an important role in memory consolidation,^30^ and therefore could have affected ALF scores and confounded the observed associations.

In summary, we have shown that the development of ALF in presymptomatic ADAD is strongly associated specifically with the thickness of the PFC, with no evidence of an association with the thickness or volume of MTL structures or other brain regions. Our results suggest that early subtle pathological changes in PFC, prior to macrostructural atrophy, may underpin ALF’s initial development, thereby highlighting the central role of PFC dysfunction in very early AD-related cognitive decline. The findings also advance our understanding of memory consolidation more broadly, pointing to ALF—potentially the earliest measurable cognitive change in AD—representing a qualitatively different (i.e. non-MTL driven) form of forgetting compared with the short interval forgetting that develops at later disease stages.

## Supplementary Material

fcaf478_Supplementary_Data

## Data Availability

Pseudo-anonymized participant data is available to share upon reasonable request. Data analysis code is available at https://github.com/csparker/parker-braincomms25-neuroanatomicalcorrelates.
