# Supplementary material for: Accelerated forgetting in presymptomatic Alzheimer’s: mediation by prefrontal cortical degeneration
Source: Brain Commun. 2025 Dec 9;7(6):fcaf478. doi: 10.1093/braincomms/fcaf478 (PMC12704326; doi:10.1093/braincomms/fcaf478)
Supplement: fcaf478_Supplementary_Data [file fcaf478_supplementary_data.docx]

**Long-term forgetting assessment methodology**

Long-term forgetting was assessed using test materials from the Adult Memory and Information Processing Battery (AMIPB).^1^ Participants were assessed on three tests: learning and recall of (1) a 15-item word list, (2) a short story, and (3) a complex visual figure. For the word list, participants had to learn the material to a minimum required accuracy of 80% over a minimum of four and maximum of ten trials. For the story the minimum accuracy was 80%, over a minimum of two and maximum of ten trials. For the figure, participants were first asked to copy the figure as accurately as possible on to a separate piece of paper; the figure and copy were then removed, and they were asked to draw the figure again from memory. Participants were then tested on free recall of the word list, story and figure, 30 minutes after presentation of the last learning trial for each test.

There were two versions of each test. Participants were randomly assigned to either set one or set two, except for families from which two members were participating, in which case the second participant in a family was always assessed on a different version from the first participant. This procedure was designed to prevent stimuli rehearsal within families during the retention interval. Participants were requested not to discuss the tests with other participants.

Following this assessment participants were given an envelope, which they were asked not to open until asked to do so during a follow-up telephone call seven days later. They were not told that the memory tests would be repeated. At the seven-day telephone call participants’ free recall of the test materials was reassessed: for the figure they were asked to draw the figure on a blank piece of paper included in the envelope; for the verbal tests this was done orally. Figure assessments were returned in a stamped addressed envelope. In addition to assessing seven-day recall and recognition raw scores, we calculated the seven-day recall as a proportion of the 30-minute recall to assess retention of information between 30 minutes and seven days.

**References**

1. Alvarez P, Squire LR. Memory consolidation and the medial temporal lobe: a simple network model. Proceedings of the National Academy of Sciences of the United States of America 1994;91:7041-7045.

|  | **Prefrontal cortex regions** | | | | **FAD cortical signature regions** | | | | | **Other cortical** | | **Sub-cortical** | |
| --- | --- | --- | --- | --- | --- | --- | --- | --- | --- | --- | --- | --- | --- |
|  | CMFC | Pars tri. | RMFC | SFC | SMC | IPC | Prec. | SPC | EC | PCC | Parahip. | Thal. | Hippo. |
| **Mutation carriers** | | | | | | | | | | | | | |
| ALF list score | | | | | | | | | | | | | |
| Left hemisphere | 0.82  .001 | 0.60  .020 | 0.65  .011 | 0.73  .003 | 0.37  .167 | 0.34  .210 | 0.30  .282 | 0.26  .340 | 0.04  .877 | 0.34  .215 | -0.46  .088 | 0.19  .500 | -0.19  .492 |
| Right hemisphere | 0.69  .005 | 0.77  .001 | 0.62  .015 | 0.76  .002 | 0.42  .114 | 0.43  .111 | 0.26  .354 | 0.27  .334 | 0.15  .587 | 0.32  .247 | 0.42  .121 | -0.02  .938 | -0.22  .420 |
| ALF story score | | | | | | | | | | | | | |
| Left hemisphere | 0.80  .001 | 0.63  .014 | 0.72  .004 | 0.82  <.001 | 0.52  .047 | 0.41  .129 | 0.46  .087 | 0.49  .064 | -0.14  .614 | 0.37  .173 | -0.16  .569 | 0.35  .198 | 0.09  .739 |
| Right hemisphere | 0.87  <.001 | 0.84  <.001 | 0.82  .001 | 0.89  <.001 | 0.63  .013 | 0.63  .013 | 0.59  .022 | 0.44  .101 | 0.15  .594 | 0.30  .277 | 0.48  .069 | 0.13  .630 | 0.05  .853 |
| ALF figure score | | | | | | | | | | | | | |
| Left hemisphere | 0.30  .315 | 0.26  .390 | 0.32  .276 | 0.43  .143 | 0.30  .320 | 0.36  .221 | 0.38  .199 | -0.05  .857 | -0.34  .259 | 0.09  .759 | 0.16  .592 | 0.37  .213 | 0.08  .800 |
| Right hemisphere | 0.02  .957 | 0.39  .185 | 0.12  .704 | 0.07  .814 | 0.17  .573 | 0.35  .243 | -0.02  .957 | -0.15  .612 | 0.07  .814 | 0.07  .814 | 0.32  .276 | 0.41  .160 | 0.16  .599 |
| **Mutation non-carriers** | | | | | | | | | | | | | |
| ALF list score | | | | | | | | | | | | | |
| Left hemisphere | 0.28  .400 | -0.09  .797 | 0.41  .206 | -0.02  .957 | 0.27  .418 | 0.31  .348 | -0.08  .818 | 0.21  .530 | 0.57  .068 | 0.11  .735 | -0.27  .418 | -0.22  .512 | 0.07  .844 |
| Right hemisphere | 0.26  .434 | -0.20  .548 | 0.07  .828 | 0.55  .080 | 0.32  .326 | 0.10  .776 | -0.25  .459 | 0.17  .605 | 0.58  .065 | -0.10  .766 | -0.04  .914 | -0.35  .285 | -0.21  .539 |
| ALF story score | | | | | | | | | | | | | |
| Left hemisphere | -0.11  .735 | -0.24  .479 | 0.21  .532 | -0.16  .626 | -0.24  .479 | -0.11  .746 | -0.15  .665 | -0.16  .626 | 0.75  .009 | -0.05  .893 | -0.59  .057 | -0.23  .500 | -0.08  .808 |
| Right hemisphere | 0.07  .829 | -0.17  .607 | -0.20  .550 | 0.31  .356 | 0.10  .767 | -0.09  .787 | -0.36  .268 | -0.06  .850 | 0.28  .396 | -0.20  .550 | -0.17  .607 | -0.53  .096 | -0.27  .412 |
| ALF figure score | | | | | | | | | | | | | |
| Left hemisphere | -0.52  .153 | -0.03  .931 | -0.18  .631 | -0.22  .569 | -0.60  .089 | -0.38  .303 | -0.10  .794 | -0.30  .426 | 0.30  .426 | -0.02  .965 | -0.37  .326 | 0.38  .303 | 0.32  .398 |
| Right hemisphere | -0.13  .728 | -0.05  .897 | -0.18  .631 | -0.26  .494 | -0.20  .600 | -0.17  .663 | -0.57  .112 | -0.33  .374 | -0.33  .374 | -0.03  .931 | -0.32  .400 | 0.35  .350 | 0.30  .426 |

**Supplementary Table 1. Association between accelerated long-term forgetting score and the thickness/volume in grey matter regions of interest.**

The top value in each box is the Spearman’s correlation coefficient with the bottom value the associated p-value. CMFC = caudal medical frontal cortex, PT = part triangularis, RMFC = rostral medial frontal cortex, SFC = superior frontal cortex, SMC = supramarginal cortex, IPC = inferior parietal cortex, prec. = precuneus, SPC = superior frontal cortex, EC = entorhinal cortex, PCC = posterior cingulate cortex, parahip. = parahippocampus, thal. = thalamus, hippo. = hippocampus.

|  | **ATR** | **CC** | **CG** | **FX** | **IOF** | **SLF II** | **UF** | **T-PREF** | **ST-FO** | **ST-PREF** |
| --- | --- | --- | --- | --- | --- | --- | --- | --- | --- | --- |
| **Mutation carriers** | | | | | | | | | | |
| ALF list score | | | | | | | | | | |
| Left hemisphere | 0.43  .108 | 0.18  .525 | 0.55  .035 | 0.41  .128 | 0.46  .085 | 0.23  .412 | 0.41  .132 | 0.44  .102 | 0.19  .483 | 0.53  .044 |
| Right hemisphere | 0.35  .195 |  | 0.34  .215 | 0.51  .055 | 0.42  .114 | 0.42  .114 | 0.47  .077 | 0.47  .077 | 0.31  .259 | 0.55  .037 |
| ALF story score | | | | | | | | | | |
| Left hemisphere | 0.27  .327 | <0.01  .995 | 0.35  .196 | 0.21  .442 | 0.25  .357 | 0.06  .833 | 0.27  .320 | 0.30  .282 | 0.06  .838 | 0.34  .208 |
| Right hemisphere | 0.01  .959 |  | 0.19  .503 | 0.27  .333 | 0.21  .457 | 0.16  .576 | 0.15  .598 | 0.16  .558 | 0.01  .959 | 0.25  .357 |
| ALF figure score | | | | | | | | | | |
| Left hemisphere | -0.02  .942 | 0.13  .677 | 0.11  .717 | 0.40  .178 | -0.02  .942 | 0.04  .892 | 0.11  .710 | 0.06  .835 | 0.08  .800 | 0.23  .443 |
| Right hemisphere | -0.32  .288 |  | 0.20  .506 | 0.37  .212 | -0.51  .073 | -0.03  .914 | -0.22  .465 | -0.02  .957 | -0.14  .650 | 0.04  .892 |
| **Mutation non-carriers** | | | | | | | | | | |
| ALF list score | | | | | | | | | | |
| Left hemisphere | -0.21  .539 | -0.29  .378 | -0.12  .724 | 0.25  .451 | -0.10  .776 | -0.20  .558 | 0.11  .745 | -0.31  .348 | 0.10  .766 | -0.29  .378 |
| Right hemisphere | -0.41  .206 |  | -0.41  .206 | 0.11  .735 | -0.21  .521 | -0.35  .291 | -0.26  .442 | -0.42  .190 | -0.04  .903 | -0.40  .223 |
| ALF story score | | | | | | | | | | |
| Left hemisphere | -0.48  .133 | -0.42  .198 | -0.32  .335 | 0.10  .767 | -0.29  .380 | -0.35  .280 | -0.11  .746 | -0.55  .078 | -0.10  .767 | -0.52  .103 |
| Right hemisphere | -0.21  .532 |  | -0.18  .587 | 0.04  .914 | -0.37  .255 | -0.12  .725 | -0.32  .335 | -0.07  .829 | -0.11  .746 | -0.10  .767 |
| ALF figure score | | | | | | | | | | |
| Left hemisphere | -0.30  .426 | -0.30  .426 | 0.00  >0.99 | -0.25  .510 | -0.50  .168 | -0.35  .350 | -0.43  .240 | -0.20  .600 | -0.25  .510 | -0.10  .794 |
| Right hemisphere | -0.57  .112 |  | -0.38  .303 | -0.13  .728 | -0.55  .124 | -0.50  .168 | -0.48  .185 | -0.27  .481 | -0.32  .400 | -0.45  .220 |

**Supplementary Table 2. Association between accelerated long-term forgetting score and the streamline density in white matter tracts of interest.**

The top value in each box is the Spearman’s correlation coefficient with the bottom value the associated p-value. ATR = anterior thalamic radiation; CC = corpus collosum (genu); CG = cingulum; FX = fornix; IOF = inferior occipito-frontal fascicle; SLF II = superior longitudinal fascicle II; uncinate fascicle; T-PREF = thalamo-prefrontal; STFO = Striato-fronto-orbital; ST-PREF = Striato-prefrontal.

| **Brain region** | **Left hemisphere** | | | **Right hemisphere** | | |
| --- | --- | --- | --- | --- | --- | --- |
|  | **Mutation carriers**  **(median, IQR)*** | **Mutation non-carriers**  **(median, IQR)*** | **Mann-Whitney U test** | **Mutation carriers**  **(median, IQR)*** | **Mutation non-carriers**  **(median, IQR)*** | **Mann-Whitney U test** |
| CMFC | 2.60 (2.52-2.67) | 2.55 (2.49-2.67) | z=-1.09  p=.287 | 2.50 (2.44-2.67) | 2.46 (2.37-2.53) | z=-1.43  p=.164 |
| PT | 2.48 (2.40-2.70) | 2.49 (2.45-2.51) | z=-0.08  p=.959 | 2.40 (2.38-2.52) | 2.40 (2.33-2.48) | z=-0.649  p=.531 |
| RMFC | 2.36 (2.31-2.47) | 2.38 (2.31-2.45) | z=0.05  p=.970 | 2.23 (2.16-2.30) | 2.23 (2.11-2.28) | z=-0.16  p=.889 |
| SFC | 2.70 (2.60-2.87) | 2.71 (2.63-2.81) | z=-0.03  p=.990 | 2.66 (2.54-2.79) | 2.66 (2.58-2.67) | z=-0.08  p=.949 |

**Supplementary Table 3. Cortical thickness in mutation carriers and non-carriers in prefrontal cortex regions of interest.**

No group differences were found between mutation carriers and non-carriers. Units are mm.

|  | **Mutation carriers** | **Non-carriers** |
| --- | --- | --- |
| **30-minute recall** |  |  |
| List 30-minute recall | 76.7 (66.7 to 80.0) | 80.0 (73.3 to 86.7) |
| Story 30-minute recall | 83.6 (80.0 to 87.5) | 87.5 (80.4 to 91.4) |
| Figure 30-minute recall | 89.7 (72.4 to 96.2) | 91.2 (78.9 to 97.4) |
| **7-day recall** |  |  |
| List 7-day recall | 30.0 (20.0 to 46.7) | 56.7 (50.0 to 63.3) |
| Story 7-day recall | 56.8 (50.0 to 69.6) | 76.0 (70.5 to 84.6) |
| Figure 7-day recall | 59.2 (50.0 to 74.9) | 76.3 (65.0 to 88.8) |
| **7-day recall / 30-minute recall** |  |  |
| List 7-day recall / 30-minute recall | 43.1 (30.0 to 61.5) | 71.8 (58.5 to 81.8) |
| Story 7-day recall / 30-minute recall | 66.7 (58.8 to 83.0) | 90.7 (78.8 to 96.7) |
| Figure 7-day recall / 30-minute recall | 72.4 (62.7 to 83.9) | 87.6 (75.7 to 96.2) |
|  |  |  |

**Supplementary Table 4. Group scores for 7-day recall, 30-minute, and the ratio of the two (i.e. the ALF score).**
